# The archaeal RNA polymerase subunit P and the eukaryotic polymerase subunit Rpb12 are interchangeable *in vivo* and *in vitro*

**DOI:** 10.1111/j.1365-2958.2008.06577.x

**Published:** 2009-01-13

**Authors:** Christoph Reich, Mirijam Zeller, Philipp Milkereit, Winfried Hausner, Patrick Cramer, Herbert Tschochner, Michael Thomm

**Affiliations:** 1 Lehrstuhl für Mikrobiologie, Universität RegensburgRegensburg, Germany; 2 Lehrstuhl für Biochemie III, Universität RegensburgRegensburg, Germany; 3 Gene Center Munich and Center for integrated Protein Science CiPSM, Department of Chemistry and Biochemistry, Ludwig-Maximilian-University MünchenMünchen, Germany

## Abstract

The general subunit of all three eukaryotic RNA polymerases, Rpb12, and subunit P of the archaeal enzyme show sequence similarities in their N-terminal zinc ribbon and some highly conserved residues in the C-terminus. We report here that archaeal subunit P under the control of a strong yeast promoter could complement the lethal phenotype of a *RPB12* deletion mutant and that subunit Rpb12 from yeast can functionally replace subunit P during reconstitution of the archaeal RNA polymerase. The ΔP enzyme is unable to form stable open complexes, but can efficiently extend a dinucleotide on a premelted template or RNA on an elongation scaffold. This suggests that subunit P is directly or indirectly involved in promoter opening. The activity of the ΔP enzyme can be rescued by the addition of Rpb12 or subunit P to transcription reactions. Mutation of cysteine residues in the zinc ribbon impair the activity of the enzyme in several assays and this mutated form of P is rapidly replaced by wild-type P in transcription reactions. The conserved zinc ribbon in the N-terminus seems to be important for proper interaction of the complete subunit with other RNA polymerase subunits and a 17-amino-acid C-terminal peptide is sufficient to support all basic RNA polymerase functions *in vitro*.

## Introduction

**Fig.1:**
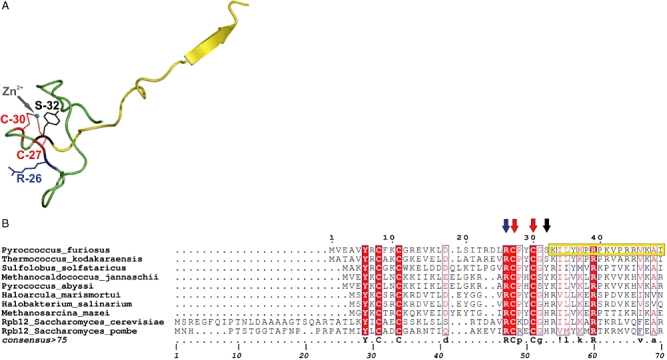
Structure and conserved sequence elements in the RNAP subunit P A. ribbon model of subunit P from *Sulfolobus solfataricus* (Hirata *et al*., 2008) generated using PyMOL. In the *Sulfolobus* structure the corresponding structural elements of the *Pyrococcus* subunit analysed and mutated in this study are shown with side chains and labelled by colours. A 17-amino-acid C-terminal peptide analyzed here is shown in yellow, the N-terminus in green, the cysteine residues 27 and 30 of the zinc-ribbon motif which were replaced by S are shown in red, R26 replaced by A in blue and S32 (Y in *Sulfolobus*) replaced by A in black. B. alignment of the protein sequence of P subunits from eight archaeal species and two yeast Rpb12 (Rpc10) sequences. The alignment was generated using ClustalW version 2.0 (Larkin *et al.*, 2007). The alignment was displayed using the ESPript software (Gouet *et al*., 1999). The consensus sequence displayed was calculated with Risler matrix and a similarity global score of 0.75% (uppercase is identity, lowercase is consensus level). The sequence of the C-terminal peptide analyzed in cell-free transcription assays is labelled with a yellow box and the single point mutations introduced into subunit P are indicated with arrows. The numbers in the first line indicate the *Pyrococcus furiosus* RpoP amino acid sequence from 1 to 49. The numbers in the last line shown in italics indicate the *Saccharomyces cerevisiae* Rpb12 amino acid sequence from position 1 to 70.

The eukaryotic subunit Rpb12, also designated as ABC10α (Carles *et al*., 1991; Rubbi *et al*., 1999), is common to all three nuclear RNA polymerases (RNAPs), the corresponding gene *RPB12/RPC10* is essential for growth in *Saccharomyces cerevisiae* and the lethal phenotype of a yeast *RPB12* null mutant is complemented by expression of its homologous counterparts from *S. pombe* and *Homo sapiens* (Treich *et al*., 1992; Shpakovski *et al*., 1995). In the crystal structure of RNAP II (polll) from yeast it contacts subunits Rpb2 and Rpb3 (Cramer *et al*., 2001) and in yeast two hybrid analyses an interaction of ABC10α with the C-terminal part of the second largest subunit of RNAP I (poll) was shown (Rubbi *et al*., 1999). This interaction required the integrity of a CX_2_C…CX_2_C zinc-ribbon motif which is conserved in this subunit between eukaryotes and archaea (see alignment in Fig. 1). Mutational analysis of the yeast CX_2_C…CX_2_C motif of ABC10α showed that only the first cysteine was essential for viability, whereas the mutation of the other three cysteine residues in the zinc-ribbon motif resulted in temperature-sensitive strains (Rubbi *et al*., 1999). A specific mutation of the third cysteine inhibited the *in vivo* activity of all three eukaryotic RNAP at 37°C, but the specific step in the transcription cycle impaired by this mutation has not yet been resolved. The fact that it is not possible to reconstitute a functional RNAP from single subunits hampers the functional analysis of subunit Rpb12 as well as of other small subunits in eukaryotic transcription.

In contrast to Eukarya the reconstitution of functional archaeal RNAPs from single subunits (Werner and Weinzierl, 2002; Naji *et al*., 2007) is possible. Furthermore, the comparison of RNAP structures by X-ray crystallography and cryo electron microscopy revealed a common architecture of eukaryotic polII and archaeal RNAPs (Cramer *et al*., 2001; Hirata *et al*., 2008; Kusser *et al*., 2008). Therefore reconstitution experiments are a useful tool to elucidate the functional importance of structural elements common to archaeal and eukaryotic RNAPs during various steps of transcription (Werner and Weinzierl, 2002; Naji *et al*., 2008; Nottebaum *et al*., 2008). In the case of the archaeal orthologue of subunit Rpb12, subunit P, such experiments revealed that an archaeal RNAP reconstituted in the absence of subunit P was greatly impaired in promoter non-specific transcription assays (Werner and Weinzierl, 2002). The ΔP enzyme assembled in a manner similar to the complete reconstituted enzyme suggesting that the contacts of subunit P with other RNAP subunits are not essential for the structural integrity of the enzyme. Nevertheless, far-Western blot analyses, binding of subunit P to subunit D immobilized via a His_6_-tag on a Ni^2+^-NTA column and structural data of the *Sulfolobus* RNAP provide strong evidence that subunit P interacts with the corresponding archaeal subunits D and B in the same manner as the eukaryotic counterpart Rpb12 with Rpb3 and Rpb2 (Goede *et al*., 2006; Hirata *et al*., 2008).

In this study, we show that the archaeal subunit P from the hyperthermophilic organism *Pyrococcus furiosus* can *in vivo* complement the essential function of subunit Rpb12 from yeast. Furthermore, Rpb12 from yeast assembles with 10 archaeal subunits to an enzyme functional in potassium permanganate footprinting assays monitoring open complex formation and in single round run-off assays.

## Results

### The archaeal subunit P complements for the essential function of Rpb12 *in vivo*

To investigate whether subunit P can functionally replace Rpb12 we used a yeast mutant with a chromosomal deletion of *RPB12* carrying the *RPB12* gene on a plasmid encoding the *URA3* marker (strain YGVS019, Shpakovski *et al*., 1995, see *Experimental procedures*). We cloned the coding region for subunit P from the hyperthermophile *P. furiosus* and from the mesophilic archaeon *Methanosarcina mazei* fused with 200 bp *RPB12* promoter and terminator region in the centromeric vector pRS314. Upon transformation of these constructs in YGVS019 and counterselection on FOA against the wt allele, growth was monitored at four temperatures from 18 to 37°C (see Fig. S1). Only constructs containing *RPB12* but not the archaeal alleles supported growth at all temperatures.

**Fig.2:**
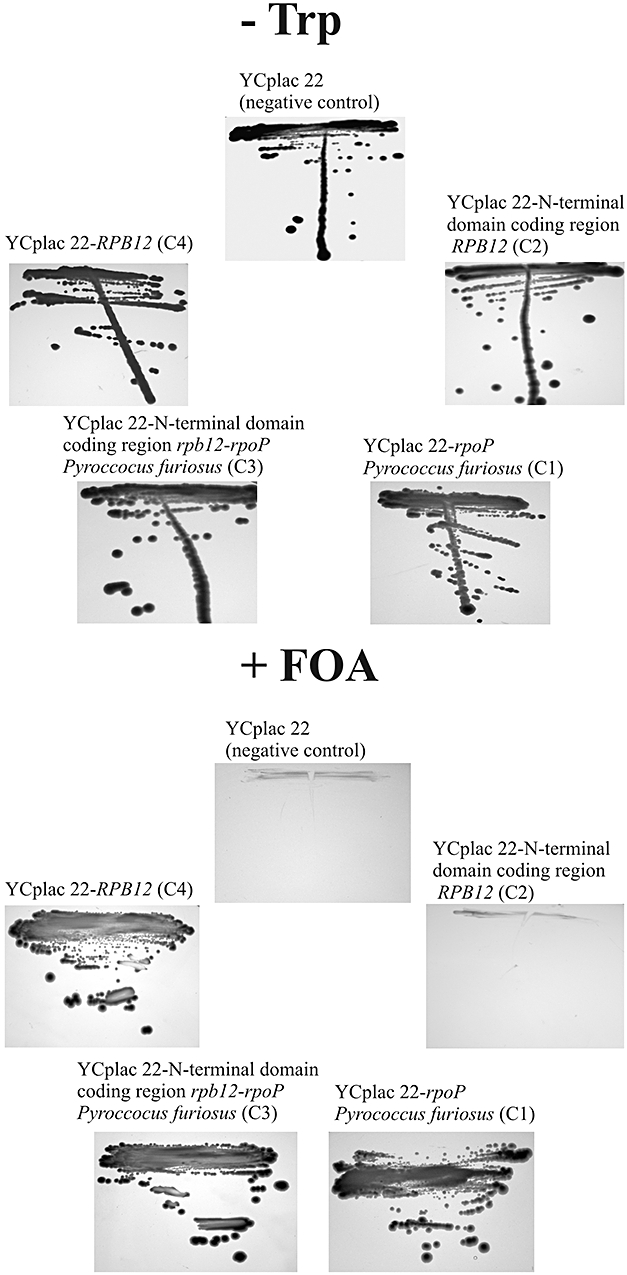
Archaeal *rpoP* can complement the essential function of yeast *RPB12.* DNA regions encoding RpoP of *Pyrococcus furiosus* (C1) were fused with the strong yeast *RPS28B* promoter and inserted in the centromeric plasmid YCplac22. The resulting constructs were transformed into yeast strain YGVS019 carrying chromosomal deletion of *RPB12* rescued by a *RPB12*/*URA3* plasmid (pFL44-*RPC10*). Transformants were transferred on 5-fluoroorotic acid containing plates to counterselect against plasmid pFL44-*RPC10* and plates were incubated at 25°C for 5 days. The cells expressing RpoP (C1) showed growth comparable to cells expressing yeast *RPB12* (C4) at 25°C and reduced growth at 37°C (data not shown). A similar construct with a chimeric gene encoding for the N-terminal domain of Rpb12 fused with *rpoP* of *Pyrococcus* (C3) could also complement the essential function of *RPB12* and did not lead to temperature sensitive growth phenotype. The N-terminal domain coding region of *RPB12* alone (C2) could not complement the essential functions of *RPB12*.

However, when we cloned the DNA region encoding for subunit P from *P. furiosus* under the control of the strong *RPS28B* promoter (C1) (Ferreira-Cerca *et al*., 2007) into the centromeric plasmid YCplac22 carrying a *TRP1* marker, cells expressing only the archaeal subunit showed normal growth at 25°C (Fig. 2) and a reduced growth rate at 37°C (data not shown). Furthermore, chimeric constructs encoding for a fusion of subunit P with the N-terminal domain of Rpb12 under the control of the above-mentioned strong promoter (C3) showed the same growth phenotype as the control strain carrying *RPB12* (C4). This finding indicates that the archaeal subunit P can functionally replace subunit Rpb12 in yeast.

### Characterization of the archaeal *Δ*P enzyme

**Fig.3:**
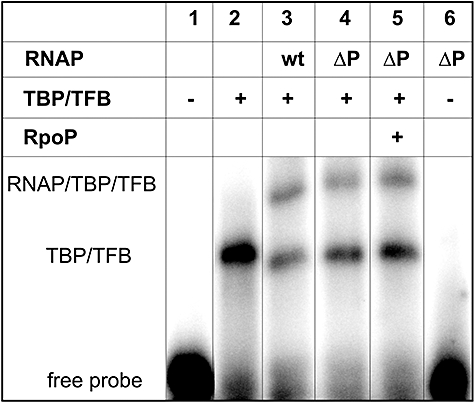
ΔP RNAP forms stable preinitiation complexes EMSA assays with a probe containing the *Pyrococcus GDH* promoter (Goede *et al*., 2006) performed in the presence and absence of TBP, TFB and reconstituted RNAP as indicated on top of the lanes. Lane 1 shows the free probe and lane 2 the TBP/TFB shift. In lane 3 the complex consisting of TBP, TFB and reconstituted wt RNAP is shown, lane 4 shows the shift formed by ΔP enzyme with TBP, TFB and in lane 5 binding reactions containing in addition 70 nM subunit P were analysed.

**Fig. 4:**
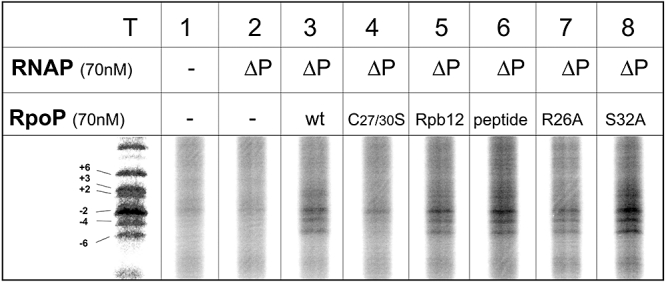
Subunit P is required for open complex formation A. DNA opening by reconstituted ΔP RNAP in the presence and absence of wild type and mutated forms of subunit P and of Rpb12 as indicated on top of the lanes was analyzed in permanganate footprinting assays as described previously (Grünberg *et al*., 2007). Positions of reactive thymidine residues were determined by a sequence standard with a labelled primer in the presence of ddTTP (T).

Previous analyses revealed a strong defect of the ΔP polymerase in promoter non-specific transcription assays (Werner and Weinzierl, 2002; S. Naji, M. Thomm, unpubl. data). To identify the steps of the transcription cycle impaired by the absence of subunit P, the ΔP enzyme was analysed in gel shift, permanganate footprinting, run-off and abortive transcription reactions and on a preformed elongation scaffold according to Kireeva *et al*. (2000). When compared with the reconstituted wt enzyme the ΔP polymerase was able to form stable complexes with promoter bound TBP-TFB during gel shifts (Fig. 3, lane 4) but did not form a potassium permanganate sensitive transcription bubble (Fig. 4, lane 2). This finding indicates that the absence of subunit P did not impair promoter recruitment of RNAP but greatly inhibited its ability to catalyse open complex formation.

**Fig. 5:**
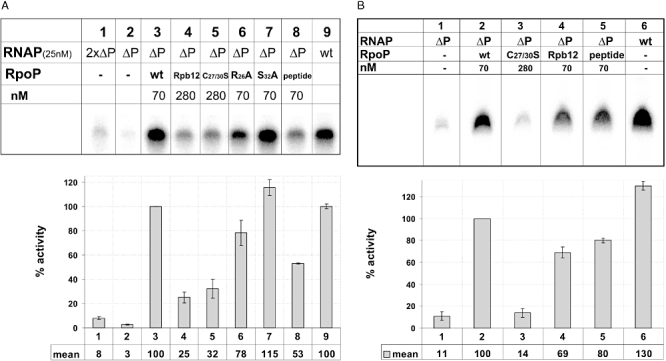
Analysis of mutant variants of subunit P and of Rpb12 in multiple round and single round run-off assays A. multiple round assays. The synthesis of a 113 nt run-off transcript from the *GDH* promoter was analyzed in standard multiple round transcription assays (see Experimental procedures) on a 6% denaturing PA gel. In lane 1 to 9 the ΔP enzyme was assayed in the presence of Rpb12 subunits or in the presence of subunit P variants in reactions containing the amounts of protein indicated on top of the lanes. Lane 10 shows the control with a reconstituted wt enzyme. The diagram below shows the mean value of the transcriptional activity. The quantification was done with the Aida image analyzer software version 3.28. B. single round assays. Ternary complexes were stalled at +20 and resumption of transcription by stalled RNAP determined as described previously (Spitalny and Thomm, 2003; 2008). The Figure shows the run-off product synthesized after addition of a complete set of unlabelled NTPs to the stalled labelled complexes (the complexes stalled at position +20 are shown in Fig. S4). In lanes 1 to 5 activity of the ΔP enzyme was determined in the presence of isolated subunit added to transcription reactions as indicated in nmoles on top. Lane 6 shows the control with a wt reconstituted enzyme. Reactions were analysed and quantified as described in A.

Analysis of multiple round run-off transcripts revealed that the ΔP polymerase is still able to synthesize small amounts of full-length RNA product in particular at higher concentrations of the enzyme (see Fig. 5A, lanes 2 and 1). The ability of ΔP to synthesize run-off transcripts was completely rescued when the isolated subunit P was added to transcription reactions (Fig. 5A, lane 3). This finding indicates that subunit P can be incorporated into the fully assembled ΔP RNAP during transcription reactions and can restore the activity defects of the ΔP enzyme.

**Fig. 6:**
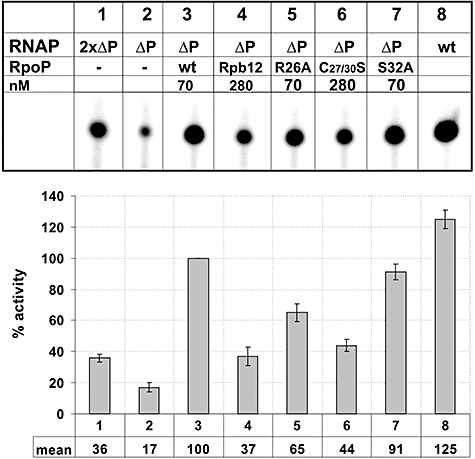
Analysis of mutant variants of subunit P and of Rpb12 in an abortive assay The ability of the enzymes to synthesize a 3 nt transcript in the presence and absence of wt P and Rpb12 and mutant variants of P was analyzed. In lanes 1 and 2 the activity of ΔP is displayed, in lane 1 twofold amount of enzyme was used. In lanes 3 to 7 subunit P, Rpb12 or P variants were added to reactions as indicated on top of the Figure. Lane 8 shows the activity of the wt reconstituted enzyme. The RNA was analyzed on a 28% PA gel. The diagram shows the mean value of transcriptional activity. The quantification was done as described in Fig. 5A.

In abortive assays measuring the synthesis of a trinucleotide from GpU (Naji *et al*., 2008) the ΔP enzyme showed ˜17% of the activity of the wt enzyme and this activity could be increased to ˜36% of wt levels when the amount of ΔP was doubled (Fig. 6, lanes 1–3). It is possible that the dinucleotide present in the abortive assay stabilizes a week or improper formed open complex and therefore contributes to a higher activity of the ΔP enzyme in this assay than in the run-off assay. Furthermore, an additional role of subunit P in steps following the synthesis of the first phosphodiester bond would also result in a higher activity of the ΔP enzyme in the abortive assay.

**Fig. 7:**
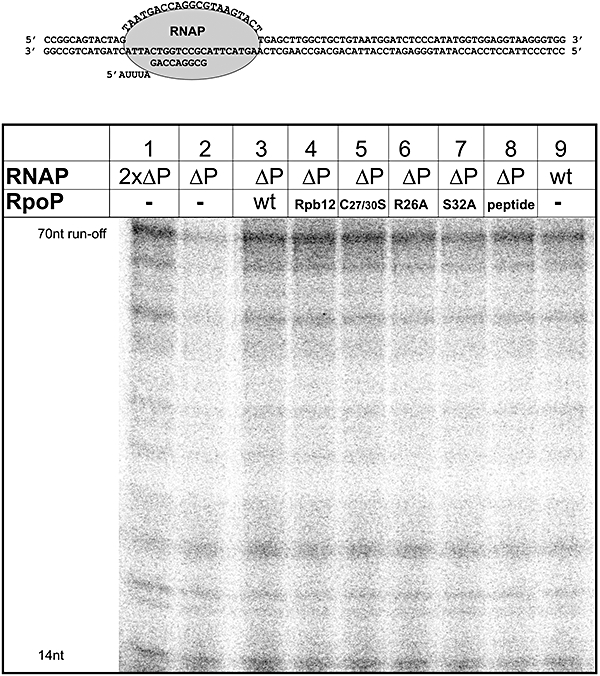
Transcription of mutant variants of subunit P and of Rpb12 on an elongation scaffold The elongation template used for this assay was assembled first by hybridization of 13 nt RNA with the template strand in the presence of RNAP at 25°C as described by Kireeva *et al*. (2000). The elongation scaffold containing both upstream and downstream duplex DNA was then completed by the addition of the complementary non-template strand. The sequence of the nucleic acids is shown on top of the Figure. In lane 1 to 8 the activity of the ΔP enzyme was determined in the presence and absence of 70 nmoles of purified P variants and of Rpb12 added to transcription reactions. Lane 9 shows the activity of the reconstituted wt enzyme on this scaffold. The diagram shows the mean value of transcriptional activity. Run-off and abortive products were quantified to get the overall activity of the enzyme on this scaffold. The quantification was done as described in Fig. 5A.

On an elongation scaffold consisting of a 13 nt RNA hybridized in part with the template strand mimicking an elongation complex (Kireeva *et al*., 2000; see schematic representation in Fig. 7) the twofold amount of ΔP enzyme compared with wt RNAP showed similar activity as the wt RNAP (Fig. 7, lanes 1–3). These findings indicate that in RNA-primed reactions the activity of ΔP RNAP is only partly impaired.

### Rbp12 can replace subunit P

When subunit P was replaced by Rpb12 during reconstitution of the archaeal enzyme, an enzyme eluting in a similar manner during Superdex 200 chromatography (Naji *et al*., 2007) as the reconstituted wt enzyme was obtained (data not shown). The RNAP reconstituted with Rpb12 showed a fourfold higher activity than ΔP in run-off transcription assays (data not shown).

When purified Rpb12 was added to multiple round run-off transcription reactions with ΔP polymerase, the activity was eightfold increased from ˜3% to ˜25% (Fig. 5A, lanes 2 and 4, and quantification of results below). As these assays were conducted for 30 min at 70°C we could not exclude that the reduced activity of Rpb12 in contrast to subunit P is mainly caused by heat denaturation of the eukaryotic Rpb12 polypeptide.

To investigate this, transcription reactions with Rpb12 were conducted for 4, 8 and 30 min and the activity of the ΔP enzyme incubated in transcription reactions in the presence of subunit P and of Rpb12 was analysed. After 4 min of incubation reactions containing Rpb12 had ˜75% of the activity of reactions conducted in the presence of P, after 8 min this ratio was ˜48% and after 30 min this ratio was only ˜30% (Fig. S2A). These data clearly demonstrate that the eukaryotic Rpb12 can in principle replace subunit P in the ΔP enzyme, but the comparison of both proteins is complicated due to the heat lability of Rpb12 derived from a mesophilic organism. As the reconstitution procedure of the RNAP from single subunits also includes a heat incubation step the reconstituted RNAP in the presence of Rpb12 resulted in a substoichiometric presence of Rpb12 in the reconstituted enzyme. This is most likely the explanation for the above-mentioned twofold reduced activity of the RNAP reconstituted in the presence of Rpb12 in comparison with the ΔP enzyme complemented with Rpb12.

For further confirmation the reconstitution of ΔP with Rpb12 or with subunit P was studied in single round assays. Briefly, in this assay RNAP was stalled on an immobilized template at +20, free and promoter bound transcription factors and free RNAP were removed by washing the complexes with a buffer containing NLS and stalled ternary complexes containing ^32^P labelled RNA were allowed to elongate the RNA to the end of the immobilized template by the addition of a complete set of unlabelled NTPs as described previously (Spitalny and Thomm, 2003). Analysis of the run-off transcripts formed in these single round assays showed that Rpb12 containing reactions had ˜70% of the activity of reactions containing subunit P (Fig. 5B, lanes 2 and 4). These data corroborate that subunit Rbp12 can functionally interact with the subunits of the archaeal enzyme and can replace subunit P.

This conclusion was further supported by permanganate footprinting assays. These assays were conducted for 5 min at 70°C. The ΔP enzyme showed only background levels of activity in this assay but a significant footprint was observed when subunit P was added to reactions with ΔP (Fig. 4, lanes 2 and 3). When Rpb12 was added to footprinting reactions with ΔP the permanganate sensitivity of the DNA in the promoter region was increased to wt levels (Fig. 4, compare lanes 3 and 5). This finding indicates that Rpb12 can also functionally replace subunit P in this assay. This was also the case in elongation assays (Fig. 7, lane 4). Taken together, these data clearly demonstrate that in principle the yeast Rpb12 subunit can carry over the role of the archaeal subunit P, but the functional analysis is hampered by the different heat stabilities of Rbp12 from a mesophile and subunit P from a hyperthermophile.

### Mutational analysis of subunit P

The N-terminal part of subunit P contains a highly conserved zinc-ribbon motif and a highly conserved R residue at position 26 (see Fig. 1A and B). The two cysteine residues at position 27 and 30 were replaced by serine to generate a zinc-ribbon minus mutant. In addition, the conserved R residue located at position 26 was replaced by alanine and the not conserved residue S32 by alanine as a control. We used a heat treatment step (10 min at 70°C) for the purification of the bacterially expressed wt and mutant forms of subunit P for purification of the recombinant polypeptides from the crude extract (see *Experimental procedures*). All the mutated subunit derivatives were stable after the heat treatment step (data not shown). In addition, all mutated subunits showed similar elution profiles like wt P when eluted from cation exchange columns (see Fig. S3A) indicating that all mutant forms of P were soluble.

Reactions containing the control mutant S32A showed a slightly increased permanganate sensitivity compared with the wt subunit P (Fig. 4, lanes 3 and 8), whereas the analysis in multiple round run-off, abortive and elongation assays exhibited a similar activity as the wt protein (Fig. 5A, lanes 3 and 7; Fig. 6, lanes 3 and 7; Fig. 7, lanes 3 and 7). This indicates that this amino acid has no significant contribution to the activity of subunit P. The analysis of the R26A mutant showed also a similar activity as the wt protein in permanganate footprinting reactions and elongation assays (Fig. 4, lanes 3 and 7; Fig. 7, lanes 3 and 6) and a weakly reduced activity in multiple round run-off and abortive assays in comparison to the wt protein (Fig. 5A, lanes 3 and 6; Fig. 6, lanes 3 and 5).

In contrast, the C27/30S mutant was severely impaired in permanganate footprinting reactions (Fig. 4, lane 4) as well as in multiple round run-off and abortive transcription assays (Fig. 5A, lane 5; Fig. 6, lane 6). To analyse whether the low activity of the zinc-ribbon mutant was caused by a lower thermal stability or whether this mutation impairs basic functions of the enzyme, the kinetics of inactivation of this mutant was determined in multiple round run-off assays and it was also analysed in single round assays. The activity detected in multiple round run-off reactions containing the C27/30S mutant was ˜35% of reactions containing wt P both after 8 and 30 min of incubation (Fig. S2B, lane 2). This finding suggests that the mutant has a similar stability as wt P but that the mutant is functionally impaired. Analysis of this mutant in single round assays revealed that reactions containing this mutant had only 14% of the activity of reactions containing wt P (Fig. 5B, lanes 2 and 3). This finding indicates again a severe functional defect of the zinc-ribbon mutant.

To further elucidate the function of this motif and to study the impact of the C-terminal part of subunit P on RNAP assembly and function we also analysed whether a soluble synthesized peptide containing residues 32–49 of subunit P (indicated in yellow in Fig. 1A and B) can complement for subunit P in some transcription assays with ΔP enzyme. Interestingly, the addition of the peptide to the ΔP enzyme could restore wt activity in permanganate footprinting assays (Fig. 4, lane 6). This finding demonstrates that the N-terminal part of subunit P containing the zinc-ribbon motif is not essential for open complex formation and subsequent analyses of the C27/30S mutant showed that this mutant was not properly inserted into the enzyme (see next section and *Discussion*) and that this was causing the observed defect in open complex formation.

Further analysis of the function of the 17-amino-acid (aa) peptide in combination with the ΔP enzyme revealed that reactions containing the peptide and ΔP were significantly impaired in multiple round run-off assays compared with the ΔP enzyme incubated with wt subunit P (Fig. 5A, lanes 3 and 8), whereas in single round assays the peptide could restore ˜80% of the wt activity (Fig. 5B, lanes 2 and 5). This finding suggests that the presence of the C-terminal peptide is sufficient to support efficient synthesis of run-off transcripts by RNAP but that the peptide seems to be similar to Rpb12 susceptible to heat denaturation. To analyse this effect in more detail the relative activity of the ΔP enzyme incubated with the C-terminal peptide was determined after 4, 8 and 30 min of incubation in transcription reactions. The relative activity compared with the enzyme incubated with wt P was ˜65% after 4 and 8 min and ˜45% after 30 min of incubation (see Fig. S2C). This result showed that the C-terminal peptide is inactivated at a higher rate than the complete subunit and this seems to account at least in part for the reduced activity in single round and multiple round run-off assays.

In contrast to the impaired multiple run-off transcription, the peptide was able to reconstitute almost wt activity in an elongation assay on a preformed scaffold (Fig. 7, lanes 3 and 8). This was also the case for the C27/30S mutant. Although this mutant is unable to catalyse open complex formation in combination with the ΔP enzyme, it could promote elongation to wt levels (Fig. 7, lanes 3 and 5). Furthermore, ‘chase experiments’ revealed that stalled transcription complexes at position +20 with the ΔP enzyme could be completely shifted by the addition of a complete set of NTPs into the run-off transcript in the presence of the peptide as well as the C27/30S mutant (Fig. S4). Taken together, these findings clearly demonstrate that the conserved N-terminal part of subunit P is not required for elongation but might play a not yet established role during the transition from initiation to elongation.

### *Δ*P RNAP is able to transcribe premelted templates

**Fig. 8:**
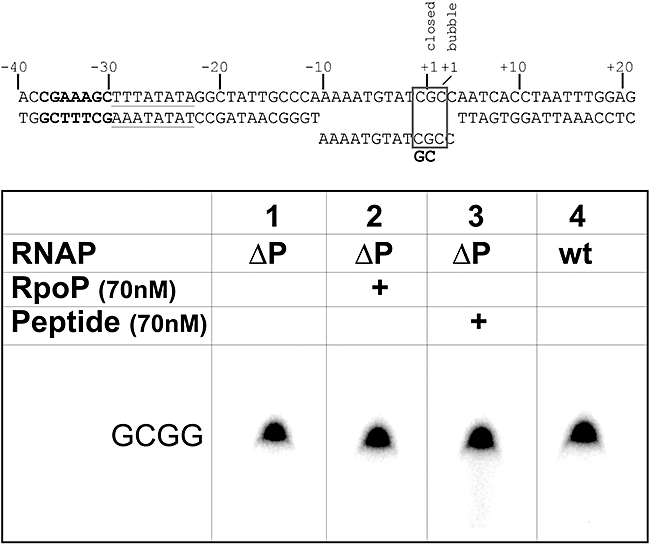
ΔP RNAP can efficiently transcribe on a preopened transcription bubble The ability of the enzymes to synthesize a 4 nt transcript in the presence of TBP and TFB on a preopened bubble was analyzed. The reaction was dinucleotide (GpC) primed and analyzed on a 24% PA gel. In lane 1 the activity of ΔP is displayed. In lane 2 wt subunit P was added to the reaction whereas is lane 3 the 17 aa peptide was used. Lane 4 shows the reconstituted wt enzyme on this scaffold as a control.

From the experiments presented so far it is clear that the C-terminal part of subunit P is essential for open complex formation (Fig. 4, lanes 2 and 6). To provide further evidence for this concept we used the ΔP enzyme on a premelted template to bypass open complex formation (Naji *et al*., 2008). Furthermore, to avoid overlaying effects due to the lack of template re-closure and thermal denaturation we measured the synthesis of a tetranucleotide from GpC for 7 min The analysis of the wt and the ΔP enzyme alone and in the presence of subunit P or the C-terminal peptide revealed a very similar activity in each assay (Fig. 8, lanes 1–4). This is additional evidence that the C-terminal part of subunit P is involved in open complex formation and the absence of subunit P does not influence the general polymerization activity of the RNAP which also indicates a kind of wt overall structure of the ΔP enzyme.

### The zinc-ribbon mutant of P incorporated into reconstituted RNAP is rapidly replaced by wt subunit P

**Fig. 9:**
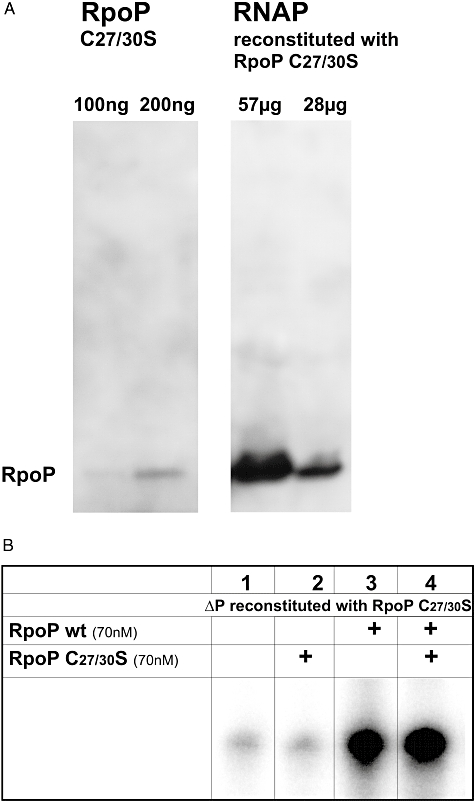
The zinc-ribbon mutant of subunit P is rapidly exchanged by the wt subunit P in the reconstituted enzyme A. the zinc-ribbon mutant form of P is contained in the Superdex purified reconstituted RNAP. A Western blot of purified subunit P (left panel) and of a Superdex 200 purified RNAP reconstituted with C27/30S is shown (right panel). The Western blot was performed with the amounts of protein indicated on top of the figure and was challenged with polyclonal antibodies against subunit P. For chemiluminescent detection of bound antibodies the Lumi-light substrate from Roche was used. B. interaction of wt P with RNAP is favoured. Run-off transcription with ΔP RNAP reconstituted with subunit P variant C27/30S. Lane 1 shows the activity of the ΔP enzyme reconstituted with subunit P variant C27/30S. The activity of this enzyme cannot be enhanced by addition of subunit P variant C27/30S in excess (see lane 2) but greatly by the addition of wt subunit P (see lane 3) indicating that wt P replaces the zinc-ribbon mutant rapidly in transcription reactions. In lane 4, both P and the zinc-ribbon mutant of P were added in excess to transcription reactions.

In contrast to the proper folding of the ΔP enzyme we believe that the tertiary structure of the zinc-ribbon mutant is distorted and that this incorrect folding is disturbing the interaction of this mutant with other subunits of RNAP. This conclusion resulted from the analysis of the single round assays: Addition of only the C-terminal peptide to ΔP RNAP resulted in a ˜5.5-fold higher activity than addition of the zinc-ribbon mutant of P (Fig. 5B, lanes 5 and 3). To investigate the interaction of the zinc-ribbon mutant with RNAP in more detail, reconstituted RNAP containing this mutant subunit was purified by Superdex 200 chromatography. RNAP activity eluted in identical fractions as reconstituted with wt P (Fig. S3B). When Superdex 200 purified RNAP reconstituted with the C27/30 mutant of P was applied to SDS-PAGE and challenged with antibodies against subunit P a clear signal was obtained by Western blot analysis (Fig. 9A, right panel). This finding indicates that the zinc-ribbon mutant of P was incorporated into the RNAP assembly. This reconstituted RNAP containing the C27/30 mutant of P showed weak activity in run-off assays which was ˜35% higher than that of the ΔP enzyme (data not shown). This activity was not affected when the isolated C27/30 mutant polypeptide was added to transcription reactions with RNAP containing the mutant form of P (Fig. 9B, lane 2). By contrast, the addition of wt P to transcription reactions containing the enzyme reconstituted with the zinc-ribbon mutant of P dramatically activated RNAP activity (Fig. 9B, lane 3). This finding indicates that wt P rapidly replaced the zinc-ribbon mutant of P in the archaeal RNAP during transcription reactions. When the isolated wt form of P was added along with the C27/30 mutant to transcription reactions, the enzymatic activity was approximately the same as when solely wt P was added (Fig. 9B, lane 4). This result supports the conclusion that the incorporation of wt P into the RNAP is favoured and that the association of wt P induced a conformation of the enzyme that excludes exchange of the wt P by the mutated form of subunit P in the RNAP assembly.

## Discussion

### The eukaryotic subunit Rpb12 and the archaeal subunit P are exchangeable

Rbp12 is a common component of RNAPs I, II and III and can be functionally exchanged between the enzymes from yeast to human indicating that the interaction surfaces of this subunit with Rpb2 and Rpb3 are highly conserved (Shpakovski *et al*., 1995). The data presented here demonstrate that the major structural determinants required for proper insertion into RNAP are also conserved between the archaeal orthologue of Rpb12, subunit P, and the eukaryotic polypeptide. At moderate growth temperature and under the control of a strong promoter the archaeal subunit P (C1) could completely rescue the lethal effect caused by deletion of the gene encoding Rpb12 (see Fig. 2). The reduced growth rate at higher temperature could be abolished by a fusion construct between the eukaryotic specific N-terminal extension and subunit P (C3). The finding of a reduced growth rate at high temperature in the absence of the N-terminal extension is in agreement with a mutational analysis of Rpb12 in yeast (Rubbi *et al*., 1999). Deletions removing the first 5 or 10 amino acids of the N-terminus show also a slight growth defect at high temperature.

Beside the activity of subunit P in yeast we also found that the eukaryotic Rpb12 can be inserted into the archaeal enzyme during reconstitution of the archaeal enzyme from single subunits (data not shown). In addition, it stimulates the activity of the reconstituted archaeal ΔP enzyme in single round assays (Fig. 5B) and in elongation assays on preformed elongation scaffolds (Fig. 7). Furthermore, the severe defect of the archaeal ΔP enzyme in open complex formation can be rescued by Rpb12 (Fig. 4). These findings indicate that the eukaryotic polypeptide can support the function of the archaeal ΔP enzyme during several crucial steps of the transcription cycle. A similar functional interaction of a eukaryotic polypeptide with the archaeal RNAP was shown for human and yeast TBP which both can functionally replace archaeal TBP in DNA-binding and run-off transcription assays (Wettach *et al*., 1995).

### Subunit P is not required for RNAP assembly but seems to be essential for open complex formation

Our studies provide also new insights into the role of Rpb12/P for RNAP assembly and function. Subunit P forms a stable complex *in vitro* with subunits D, L and N (Werner *et al*., 2000; Werner and Weinzierl, 2002) and also the reconstitution of a B-D-L-N-P complex has been reported (Goede *et al*., 2006). These findings and the highly specific interaction between human Rbp12 and Rpb3 (Acker *et al*., 1997) and archaeal P and D (Goede *et al*., 2006) suggested an important role of Rpb12 and P as platform for recruitment of larger subunits during RNAP assembly. It was reported that the ΔP enzyme assembled in a manner similar to the wt enzyme, but showed a strong defect in promoter non-specific transcription assays (Werner and Weinzierl, 2002). Our findings that the ΔP enzyme is unable to form an open complex as analysed by potassium permanganate footprinting (Fig. 4), but shows wt activity in abortive assays on premelted templates (Fig. 8) indicates that this subunit is directly or indirectly involved in open complex formation. The structural basis for the requirement of subunit P during open complex formation is unknown. However, subunit P binds between subunit D and the wall and protrusion domains of subunit B (Hirata *et al*., 2008), and its absence therefore likely results in an increased positional flexibility of the wall and protrusion domains. Since these domains are near the TFB/TBP complex during initiation (Hahn, 2004; Chen and Hahn, 2004; Chen *et al*., 2007), it is possible that their increased flexibility or an altered position impairs normal DNA strand separation during open complex formation.

The findings that the ΔP enzyme shows still significant activity on an elongation scaffold (Fig. 7) demonstrate that this subunit is not essential for the assembly of an RNAP capable to catalyse the elongation step of transcription. In addition, subunit P can rapidly activate ΔP to wt levels of activity when added to transcription reactions (Figs 4 and 5). This finding suggests that P can be rapidly and easily inserted into the enzyme assembled in the absence of subunit P and this finding supports the conclusion that the formation of a Rpb2–3-10-11-12 subcomplex is not a necessary step in the pathway of RNAP assembly. This also implies that Rpb12 plays no essential role for the recruitment of the two large RNAP subunits.

### The C-terminal part of P is sufficient to aid different RNAP functions

Two characteristic features of Rpb12 are a zinc-ribbon motif in the N-terminus and a conserved basic C-terminal part and two conserved arginine residues located at position 47 and 60 in the yeast enzyme (Cramer *et al*., 2001; see also Fig. 1). Only one of the four cysteine residues of yeast Rpb12 was essential for growth of yeast (Rubbi *et al*., 1999) and the mutants of the two cysteine residues corresponding to cysteine 48 and 51 in yeast showed no phenotype (Rubbi *et al*., 1999). The zinc-ribbon mutant analysed in this study showed a clear defect in permanganate footprinting reactions but several lines of evidence indicate that the zinc ribbon is not essential for open complex formation. First, addition of a truncated form of P lacking the N-terminus completely resulted in wt levels of activity in permanganate footprinting assays (Fig. 4, lane 6). Second, replacement of subunit P in the RNAP by the C-terminal peptide lacking all conserved residues with the exception of lysine37 and argine39 (see Fig. 1) and all four cysteine residues of the zinc-ribbon motif resulted in a ˜20% reduction of RNAP activity in single round run-off assays whereas a double Cys-Ser replacement on positions 27 and 30 of P caused a 86% reduction of RNAP activity (Fig. 5B, lanes 3, 5 and 6). This indicates that deletion of the entire N-terminal part containing the zinc-ribbon motif impairs activity of RNAP less than the replacement of the third and fourth cysteine residues of this motif. Third, addition of wt P to an RNAP reconstituted with C27/30 mutant form of P resulted in a dramatic increase in activity in multiple round run-off assays (Fig. 9B, lane 3). Therefore, the zinc-ribbon mutant of P seems to be only loosely associated with the enzyme and this improper association is most likely responsible for the observed defect in open complex formation. Our finding that at least low levels of run-off transcripts can be formed by the enzyme containing the zinc-ribbon mutant form of P (Fig. 5) and that elongation of transcription is not affected by this mutation (Fig. 7) indicate that transcription is not completely inhibited by this promoter opening defect. The finding of Rubbi *et al*. (1999) that a double mutation of the corresponding two cysteine residues in yeast Rpb12 (Cys48 and Cys51) resulted in a wt growth phenotype *in vivo* (Rubbi *et al*., 1999) indicates that the promoter opening defect of this mutant can be compensated *in vivo*.

The charge of the C-terminal domain of subunit P was essential for yeast *in vivo*. Alteration of the charge of this domain by amino acid replacements impaired growth strictly or was lethal (Rubbi *et al*., 1999). In our study replacement of a conserved arginine residue on position 26 (located in the N-terminal part) of subunit P had only a moderate effect on RNAP activity in various assays indicating that the charge of this residue is not important for RNAP function. Our study provides evidence that the conserved C-terminal part of subunit P is essential and sufficient for basic RNAP functions. Analysis of the peptide containing the conserved residues in the C-terminus revealed that addition of this peptide to transcription reactions containing the ΔP enzyme greatly stimulated or completely rescued RNAP activity in permanganate footprinting and single round assays and this finding suggests that this part of P is sufficient to support the major functions of RNAP in the transcription cycle.

### Evolutionary implications

The archaeal RNAP seems to be the simplified version and evolutionary precursor of the three eukaryotic RNAPs. In line with this statement the most conserved parts of the enzyme are located in the cleft comprising structural elements essential for catalytic activity like the bridge helix, the pore and the clamp and outside the active centre the dock domain and its surrounding regions (Goede *et al*., 2006; Hirata *et al*., 2008; Kusser *et al*., 2008). Many of the smaller subunits of the eukaryotic RNAP like Rpb4, Rpb5, Rpb6 and Rpb12 evolved additional N-terminal domains, which seems to be involved in eukaryotic-specific functions. But the findings that the archaeal subunit P could complement for the eukaryotic Rpb12 in yeast and Rpb12 could substitute for subunit P in an *in vitro* reconstituted RNAP clearly demonstrate that these RNAPs have a common ancestor and there is an evolutionary pressure to keep the basic structure of this type of RNAP conserved.

## Experimental procedures

### Transcription assays

*Promoter-directed run-off* in vitro *transcription assay.In vitro* transcription assays were performed as described (Naji *et al*., 2007). Standard run-off transcription reactions contained 100 ng of *GDH* promoter, 25 nM RNAP, 50 nM TFB and 60 nM TBP in a total volume of 25 µl of transcription buffer [40 mM Na-HEPES, pH 7.3, 250 mM NaCl, 2.5 mM MgCl_2_, 5% (v/v) glycerol, 0.1 mM EDTA, 5 mM β-mercaptoethanol, and 0.1 mg ml^−1^ bovine serum albumin]. NTPs were added to a concentration of 40 µM (ATP, GTP and CTP) and 2 µM UTP and [α-^32^P] UTP at 0.15 MBq (110 TBq/mmol). The components and subunit P variants were added as indicated on top of the figures and the reactions were incubated for 30 min at 70°C. RNA was extracted with phenol-chloroform, ethanol precipitated and the RNA pellet washed in ethanol and resuspended in H_2_O. Labelled transcripts were analysed by electrophoresis in 6% (w/v) polyacrylamide urea gels. The transcription products were visualized using an image plate and Image Analyzer (FLA-5000, Fuji, Japan).

### Single round transcription assays

Immobilized ternary complexes were stalled at position +20 relative to the transcription start site and were isolated according to Spitalny and Thomm (2003). To remove unbound RNAP and TBP/TFB from promoter DNA, complexes were washed with transcription buffer containing 0.1% *N*-lauroylsarcosine (NLS). Then the isolated ternary complexes were resuspended in transcription buffer and supplemented with all four nucleotides (40 µM each) but no additional radioactivity in a total volume of 25 µl. During further incubation for 3 min at 70°C run-off transcripts were formed. Transcription reactions were stopped by phenol-chloroform extraction.

### Abortive transcription assays

The transcription reactions were performed similar to run-off transcription but with a −C/+20 cassette of the *GDH* promoter according to Spitalny and Thomm (2003). The transcription was dinucleotide primed with GpU (40 µM) and transcription was started by the addition of radiolabelled UTP (3.3 pmol). The 3 nt RNA product was analysed on a 28% (w/v) polyacrylamide urea gel. To mimick an open complex a premelted template (Fig. 8) was used. In this case the transcription was performed with GpC (40 µM) and radiolabelled GTP (3.3 pmol) for 7 min at 70°C. The 4 nt RNA product was analysed on a 24% (w/v) denaturing polyacrylamide gel.

#### Transcription on an elongation scaffold

The reactions contained the same salt and NTP concentrations as the run-off transcription assays but the template was reconstituted according to Kireeva *et al*. (2000) and transcription factors were omitted from the reactions. Subunit P, Rpb12 and subunit P variants were added to a final concentration of 70 nM when the reaction was assembled at 4°C. To hybridize the template strand with RNA both components were heated for 3 min at 95°C and allowed to cool down to 20°C in the heating block overnight. After preincubation of the 83 nt T-strand and the 13 nt RNA in the presence of reconstituted RNAP for 5 min at 25°C, the complementary NT-strand was added and the incubation was continued for 5 min at 25°C (for sequence of nucleic acids see Fig. 7). Then, the reactions were incubated for 30 min at 70°C. The RNA was analysed as described on 24% (w/v) polyacrylamide urea gels.

### Electrophoretic mobility shift assay

A DNA fragment spanning the *GDH* promoter region from −60 to +37 was used as probe as described previously (Goede *et al*., 2006). The binding reactions contained 63 nM recombinant Pol, 126 nM TBP, 106 nM TFB, 50 nM *GDH* promoter and 1 µg of poly [dI-dC)] as non-specific competitor DNA. 70 nM subunit P was added as indicated on top of Fig. 3. Protein-DNA complexes were assembled in a 25 µl volume containing 40 mM Na-HEPES, pH 7.3, 250 mM NaCl, 25 mM MgCl 2, 0.1 mM EDTA, 5 mM β-mercaptoethanol, 5% (v/v) glycerol and 0.1 µg ml^−1^ bovine serum albumin.

The transcription factors were preincubated with the DNA at 70°C for 5 min. Then, the RNAP was added and reactions were incubated for 5 min at 70°C. Then, the reactions were cooled to 37°C and competitor DNA was added and the reactions were incubated for 10 min at 37°C. DNA-protein complexes were analysed on native 5% PA gels (1× TBE, 4% glycerol, 40 mM β-mercaptoethanol). After electrophoresis at 30°C the complexes were visualized by phosphoimaging.

### KMNO4 footprinting

Thymidine residues in open complexes were detected by treatment with potassium permanganate as described previously (Grünberg *et al*., 2007). Reconstituted RNAP was preincubated for 5 min at 70°C with a DNA fragment encoding the *GDH* promoter in reactions containing 500 nM TFE, 440 nM RpoE′-F, 70 nM RNAP, 60 nM TBP and 50 nM TFB. The subunit P variants were added to the footprinting reactions; the amount of subunit P in the reaction was 70 nM.

### Western blot analyses

The wt and mutant derivatives of subunit P were detected in Superdex 200 purified RNAP fractions by Western blotting as described previously (Hausner and Thomm, 1995). The proteins were separated by SDS-polyacrylamide gel electrophoresis on a 15% gel and transferred to PVDF membranes (Immobilon-P^SQ^; 0.2 µm; Millipore) by semidry blotting. The membrane was incubated with primary antibody against subunit P (1:750) and peroxidase coupled secondary antibody (1:5000) from chicken. Bound antibodies were detected with lumi-light Western blotting substrate according to the instructions of the manufacturer (Roche). The signals were visualized on Biomax light films (Kodak).

### Site-directed mutagenesis and purification of subunit P variants

*S*ite-directed mutagenesis was carried out using the Stratagene QuickChange II site-directed mutagenesis kit (Stratagene, La Jolla, CA). The following reagents were incubated: 10 µM each of the two primers, 25 ng of parental plasmid template pET17b-*rpoP* (Novagen), 0.5 µl of 10 mM dNTP mix, 2.5 µl of 10× reaction buffer, 0.5 µl of *Pfu* DNA polymerase (2.5 units µl^−1^; Stratagene, La Jolla, CA) and H_2_O to a final volume of 25 µl. All the other steps were performed as described by the Stratagene manual.

### Complementation assays, yeast strains and plasmids

For complementation assays the yeast strain YGVS019 {*MAT*a *his3*-Δ*200 lys2–801 trp1*-Δ*1 ura3–52 ade2–1 rpc10*-Δ::*HIS3*[pFL44-RPC10: 2 µ*URA3 RPC10* (Sc10a)]; Shpakovski *et al.,* 1995} was used. The coding region for RpoP of *P. furiosus* (PF 2009; C1), for the N-terminal domain of Rpb12 (P 40422; aa 1–28; C2), for a chimeric protein containing the N-terminal domain of Rpb12 (P 40422; aa 1–28) plus RpoP of *P. furiosus* (aa 6–49; C3) and for yeast Rpb12 (C4) were cloned in fusion with the yeast *RPS28B* promoter region of plasmid K520/YEplac195-pRPS28 (Ferreira-Cerca *et al*., 2007) and a yeast *RPB12* stop codon into the yeast centromeric vector YCplac22 (Gietz and Sugino, 1988). Plasmids C1-4 were transformed in strain YGVS019 carrying chromosomal deletion of *RPB12* rescued by a *RPB12*/*URA3* plasmid (pFL44-*RPC10*). The YCplac22 vector encoding *TRP1* supported growth on tryptophan-free minimal media (SDC; MP Biomedicals) and after counterselection on FOA against pFL44-*RPC10* growth was monitored at four temperatures (18, 25, 30 and 37°C) by plating on minimal media containing 5-fluoroorotic acid (1 g l^−1^; Toronto Research Chemicals) as described previously (Widlund and Davis, 2005). In the centromeric plasmid pRS314 (Sikorski and Hieter, 1989), constructs encoding for RpoP of *P. furiosus* (PF 2009) or *M. mazei* (MM 2626) fused with 200 bp promoter and terminator region from Rpb12 (Rpc10) of *S. cerevisiae* were also tested for the complementation of Rpb12 *in vivo*. The shuffle was analysed at four temperatures (18, 25, 30 and 37°C) as described above.

### Purification of subunit P variants

The subunit P variants were expressed in BL21 (DE3) CP cells (Stratagene) basically as described previously by Naji *et al*. (2008). The crude extract was heat treated at 70°C for 10 min and the subunit P variants were purified using a SP XL 1 ml column (GE Healthcare) with linear salt gradients from 10 mM to 1 M NaCl in buffer containing 40 mM Hepes-Na pH 7.6, 10% glycerol, 1 mM ZnSO_4_, 1 mM PMSF and 5 mM β-mercaptoethanol. The protein concentration was determined in a Bradford assay.

### Synthesis of the C-terminal domain of subunit P

The 17-amino-acid peptide (KILYKPRPKVPRRVKAI) was purchased from JPT Peptide Technologies, Berlin. The purity was higher than 90%.
