# Supplementary material for: The archaeal RNA polymerase subunit P and the eukaryotic polymerase subunit Rpb12 are interchangeable in vivo and in vitro
Source: Mol Microbiol. 2009 Jan 13;71(4):989–1002. doi: 10.1111/j.1365-2958.2008.06577.x (PMC2680338; doi:10.1111/j.1365-2958.2008.06577.x)
Supplement: Supplementary file 1 [file mmi0071-0989-SD1.pdf]

Fig. S1

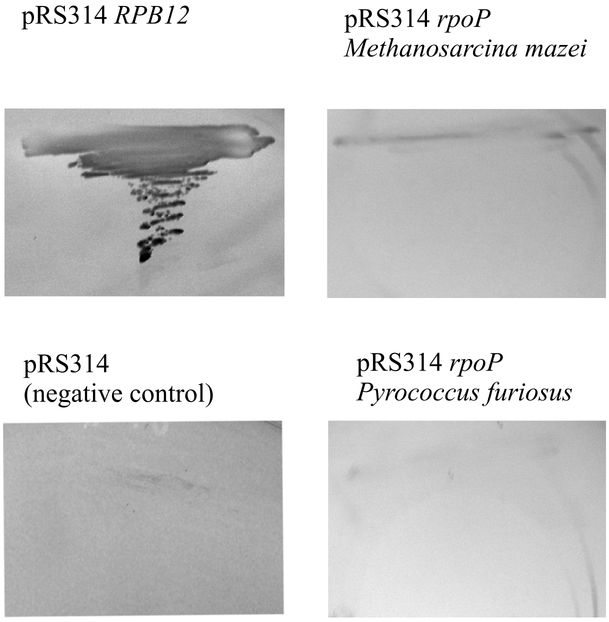

FIGURE S1. **Archaeal *rpoP* cannot complement the essential function of yeast *RPB12* under the control of the *RPB12* promotor**. DNA regions encoding RpoP of *Pyrococcus furiosus* or RpoP of *Methanosarcina mazei* fused with 200 bp *RPB12* promotor and terminator region were inserted in the centromeric plasmid pRS314. The resulting constructs were transformed into yeast strain YGVS019 carrying chromosomal deletion of *RPB12* rescued by a *RPB12*/*URA3* plasmid (pFL44-*RPC10*). Transformants were transferred on 5-fluoroorotic acid containing plates to counterselect against plasmid pFL44-*RPC10* and plates were incubated at temperatures ranging from 18 to 37 °C. The figure shows growth at 25 °C for 5 days. Only cells expressing *RPB12* showed growth at all temperatures.

Fig. S2 A

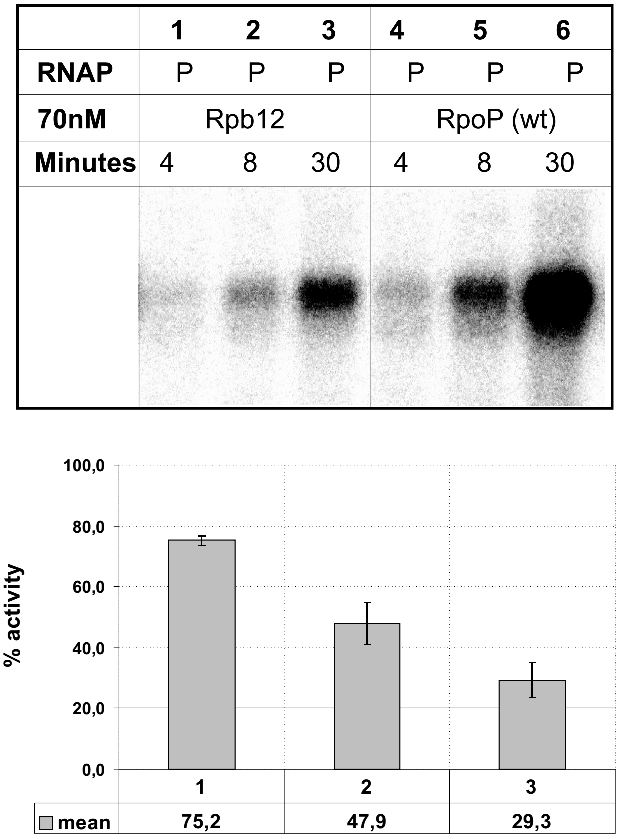

Fig. S2 B

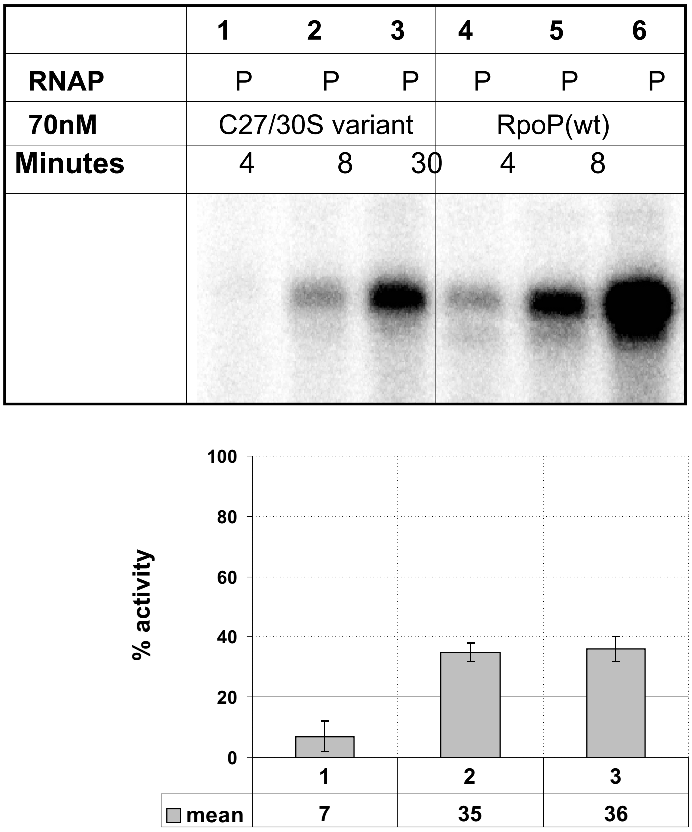

Fig. S2 C

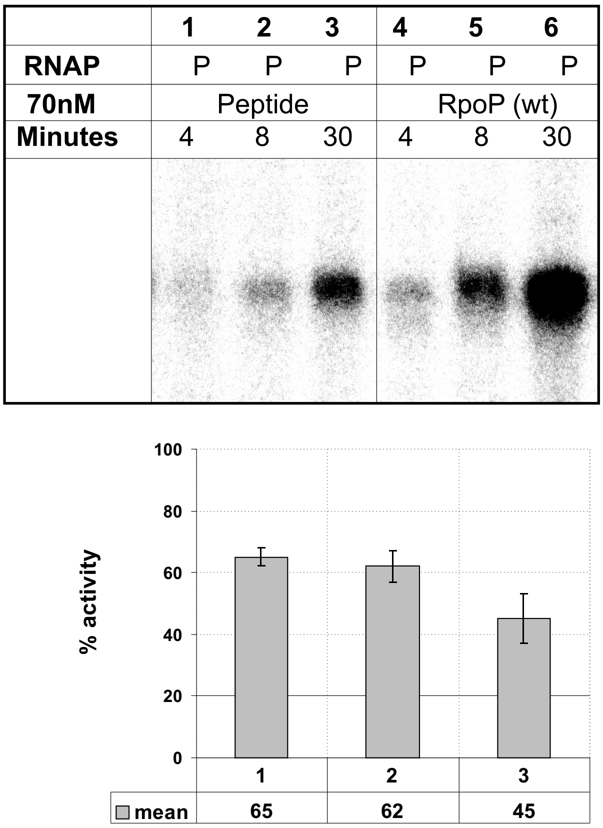

FIGURE S2. **Stability of Rpb12 and of P variants during transcription.** Run-Off transcription reactions with P RNAP were supplemented with either wt P, Rpb12 or mutant P variants and samples were taken after 4, 8 and 30 minutes and analyzed on a 8 % denaturing PA-gel (top of each panel). The amount of synthesized transcript was quantified at each time point and the transcriptional activity obtained in the presence of wt P was defined as 100 percent (diagram below the denaturing PA-gels). *A*, Relative activity of transcription reactions with P RNAP containing Rpb12 was compared with reactions containing wt P (see quantification below the Figure). *B,* reactions containing the zinc ribbon mutant C27/30S were compared with reactions containing wt P as described in *A*. *C,* The relative activity of the P RNAP incubated with the peptide was determined in comparison with reactions containing wt P was determined as described in *A*.

Fig. S3 A

Fig. S3 B

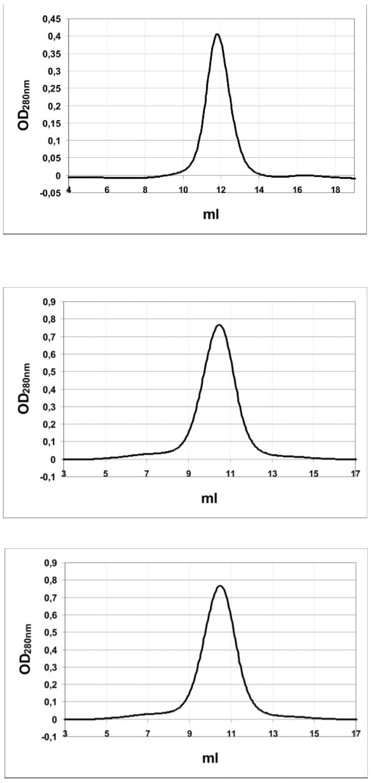

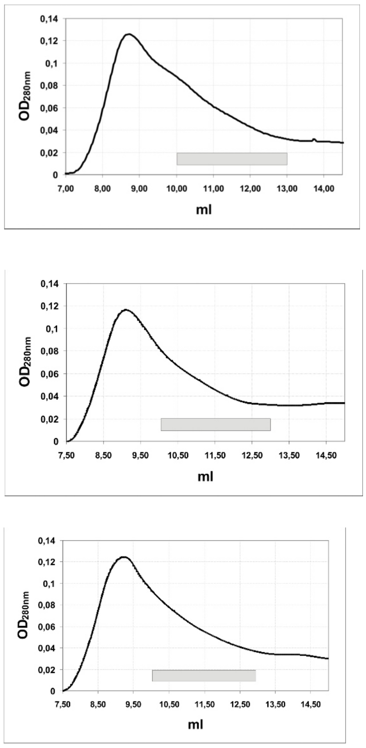

FIGURE S3. **Elution profile of P variants and of RNAP reconstituted with P variants**. *A*, the elution profile of three mutated P variants from a SP XL 1ml cation exchange chromatography column is shown. The proteins were eluted with a linear gradient ranging from 10 to 1000mM NaCl (see Experimental Procedures). In profile 1 the subunit P variant C27/30S mutant is displayed, in 2 the subunit P variant R26A and in 3 subunit P variant S32A. *B,* elution profile of RNAP reconstituted with the P zinc ribbon mutant and of wt reconstituted RNAP. The Figure shows the Superdex 200 elution profiles. The transcriptionally active fractions eluting between 10-13 ml are indicated with grey bars. In profile 1 the P RNAP is displayed, in 2 P RNAP reconstituted with subunit P C27/30S and in 3 wt *Pfu* RNAP.

Fig. S4

	1	2	3	4	5	6
RNAP	P	P	P	P	P	wt
RpoP	-	wt	C27/30S	Rpb12	peptide	-

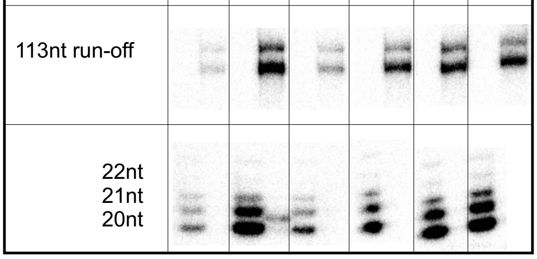

FIGURE S4. **Resumption of paused RNAPs containing mutant forms of subunit P under single round conditions**. Ternary complexes were stalled at +20, washed and the reactions divided into two aliquots. One aliquot was analyzed on a 20 percent sequencing gel (first lane of each panel). The second aliquot was chased by the addition of a complete set of non labelled NTPs as described previously (27) for 3 minutes at 70 °C (second lane in each panel). In lanes 1 to 5 the activity of the P enzyme incubated with various mutants of subunit P (70nM) as indicated on top of the lanes was analyzed. Lane 6 shows the control reaction with a reconstituted wt enzyme.
